# Surgical strategy of pituitary and cavernous sinus exploration in MRI–Negative Cushing’s disease after unsuccessful surgery

**DOI:** 10.3171/2023.4.FOCVID2312

**Published:** 2023-07-01

**Authors:** Ming Shen, Xuefei Shou, Yongfei Wang

**Affiliations:** 1 Department of Neurosurgery, Huashan Hospital, Shanghai Medical College, Fudan University, Shanghai;; 2 Neurosurgical Institute of Fudan University, Shanghai;; 3 Shanghai Clinical Medical Center of Neurosurgery, Shanghai;; 4 Shanghai Key Laboratory of Brain Function Restoration and Neural Regeneration, Shanghai;; 5 Shanghai Pituitary Tumor Center, Shanghai;; 6 National Center for Neurological Disorders, Shanghai; and; 7 Research Units of New Technologies of Micro-Endoscopy Combination in Skull Base Surgery (2018RU008), Chinese Academy of Medical Sciences, Beijing, China

**Keywords:** adenoma, Cushing’s disease, cavernous sinus, endoscopic endonasal surgery, surgical technique

## Abstract

MRI–Negative Cushing’s disease continues to be a challenging disease despite better imaging and techniques. The situation can be more complicated in the setting of prior surgery or failed surgery. Often, a narrow surgical corridor is encountered with robust cavernous or intercavernous sinuses. Controlling venous oozing properly is critical to achieving better outcomes. In this video, the authors present a case of MRI–Negative Cushing’s disease after previous unsuccessful surgery. The pituitary tumor was detected on the left side of the gland, close to the cavernous sinus. Margin-plus resection is important if it can be achieved. Biochemical remission was achieved after surgery.

The video can be found here: https://stream.cadmore.media/r10.3171/2023.4.FOCVID2312

## Transcript

### 0:20 Clinical History.

This is an illustrative case showing our surgical strategy of pituitary and cavernous sinus exploration in MRI–Negative Cushing’s disease after previous unsuccessful surgery. This 46-year-old lady presented with 10 years’ history of edema, hypertension, and central obesity. Cushing’s disease was confirmed after a series of diagnostic tests. A transsphenoidal surgery was performed in another hospital 2 years ago. Unfortunately, they did not find the tumor, and biochemical remission was not achieved. The specimen sent for pathology was proved to be gland cells. The patient was transferred to our hospital. She lost her preoperative images during the house move. On admission, the pituitary MRI with contrast could not identify any definite lesions. There were multiple low-enhancing lesions in the gland, with the largest one on the left side close to the cavernous sinus. However, the pituitary stalk was slightly toward the left side, not supporting a left-sided tumor.

### 1:23 Surgical Plan and Patient Positioning.

So our surgical plan was to explore the whole pituitary gland, including the cavernous sinuses, if necessary.^1^ Here are the key steps. First is a standard endoscopic endonasal approach to expose the sella and bilateral medial cavernous sinuses.^2^ Pack the cavernous or intercavernous sinuses if needed. Explore the left, followed by the right side of the pituitary gland toward the medial wall of the cavernous sinus. Resect the medial wall of the cavernous sinus if tumor invasion is suspected.^3^ Explore the cavernous sinuses if no tumor was found in the pituitary gland—finally, reconstruction. The patient was supine with the upper body elevated by 20° to facilitate venous drainage. The head was rotated 15° toward the operator to provide an ergonomic trajectory. HD endoscope, neuronavigation, intraoperative micro-Doppler ultrasound, and endoscopic microsurgical instruments were prepared.

### 2:22 Exposure.

Here is an intraoperative view of our exposure. As you can see, there was a scar on the right side, indicating the range of dura opening and surgical exploration of the previous surgery.

### 2:35 Pituitary Exploration: Left Side.

According to the surgical plan, we explored the left side first. Here we can see that the left cavernous and intercavernous sinuses were a little bit robust. After Doppler detection, we opened the ventral wall of the left cavernous sinus and injected hemostatic materials to control venous oozing. Now we opened the left sella dura with a sharp feather blade. The underlying orange-yellow gland tissue was partially removed. We used a small, angled pituitary curette to split the gland, carefully searching tumor-like tissues. Here we can see a gray-white bulging surface, indicating an underlying tumor. After cutting into it, we can see gray-white soft tumor-like lesions. The lesion was collected for pathological diagnosis. The intraoperative frozen section showed a pituitary tumor. We used small pituitary forceps to create an adequate space for the sucker to debulk this soft tumor. A pseudocapsule had not been formed for this tiny tumor.^4^ Enlarged resection, including millimeters of the adjacent gland, is critical for surgical cure.^1^ The inferior margin, including the dura, could be easily achieved using a microscissor. The lateral margin was quite tenacious. A healthy, clean medial wall of the cavernous sinus, which was the scheduled lateral boundary of pituitary exploration, could not be obtained using this inside-out fashion.

### 4:05 Cavernous Sinus Exploration: Left Side.

The medial wall of the cavernous sinus was dissected toward the midline, and we can see this medial wall was thick, indicating tumor infiltration. Here we should notice that the medial wall was connected to the cavernous ICA with the inferior parasellar ligament.^5^ Sharp dissection helps to protect the ICA from laceration. Another critical point to avoid carotid injury is the identification of the inferior hypophyseal artery.^5^ It should be precisely coagulated and cut. Then the medial wall of the cavernous sinus, together with the tumor remnants, could be cut as a whole, creating a tumor-free lateral margin. Afterward, we resected additional millimeters of gland tissues at the superior and medial margins. Final exploration of this side showed the resection cavity was quite satisfied. The posterior wall of the cavernous sinus and the base of the posterior clinoid process was nicely exposed. Notice that pretreating the cavernous sinus with hemostatic materials injection helps maintain a clean surgical field, increasing safety during cavernous sinus exploration.^6^

### 5:20 Pituitary Exploration: Right Side.

We explored the right side as scheduled to exclude multiple tumors at the right gland. After Doppler detection, the dura was cut toward the right cavernous sinus. The right pituitary gland was explored toward the medial wall of the cavernous sinus. Here we can see the medial wall was healthy and thin. No tumor-like tissue was identified.

### 5:43 Reconstruction.

As no intraoperative CSF leak was created, a simple reconstruction was performed using collagen matrix and Gelfoam, wedged with a piece of semirigid collagen fleece and covered with fibrin glue.^7^

### 5:56 Postoperative Course.

The patient tolerated the procedure well. Her ACTH quickly decreased to 3.5 pg/mL, and cortisol decreased to 0.7 μg/dL, indicating remission. Pathology found ACTH cell adenomas in the specimen from the left gland, and ACTH hyperplasia in the affected left medial wall of the cavernous sinus. Postoperative MRI demonstrated a nice resection cavity on the left side. Thank you.

## Acknowledgments

This work was funded by the Chinese Academy of Medical Sciences (grant no. 2019-I2M-5-008 to Y.W.) and the National Natural Science Foundation of China (grant no. 81602191 to M.S.).

## Disclosures

The authors report no conflict of interest concerning the materials or methods used in this study or the findings specified in this publication.

## Author Contributions

Primary surgeon: Wang. Assistant surgeon: Shen, Shou. Editing and drafting the video and abstract: Shen. Critically revising the work: all authors. Reviewed submitted version of the work: all authors. Approved the final version of the work on behalf of all authors: Wang. Supervision: Wang.
